# Monitoring *Leishmania* infection and exposure to *Phlebotomus perniciosus* using minimal and non-invasive canine samples

**DOI:** 10.1186/s13071-020-3993-7

**Published:** 2020-04-21

**Authors:** Carla Maia, José Cristóvão, André Pereira, Tatiana Kostalova, Tereza Lestinova, Petra Sumova, Petr Volf, Lenea Campino

**Affiliations:** 1 Global Health and Tropical Medicine (GHTM), Instituto de Higiene e Medicina Tropical (IHMT), Universidade NOVA de Lisboa (UNL), Lisbon, Portugal; 2 Medical Parasitology Unit, IHMT-UNL, Lisbon, Portugal; 3 Department of Parasitology, Faculty of Science, Charles University in Prague, Prague, Czech Republic

**Keywords:** Blood, Conjunctival cells, Dog, Exposure, *L. infantum*, *Phlebotomus pernicious*, Saliva

## Abstract

**Background:**

In endemic areas of zoonotic leishmaniosis caused by *L. infantum*, early detection of *Leishmania* infection in dogs is essential to control the dissemination of the parasite to humans. The aim of this study was to evaluate the serological and/or molecular diagnostic performance of minimally and non-invasive samples (conjunctiva cells (CS) and peripheral blood (PB)) for monitoring *Leishmania* infection/exposure to *Phlebotomus perniciosus* salivary antigens in dogs at the beginning and the end of sand fly seasonal activity (May and October, respectively) and to assess associated risks factors.

**Methods:**

A total of 208 sheltered dogs from endemic areas of leishmaniosis were screened. *Leishmania* DNA detection in PB on filter paper and CS was performed by nested-PCR (nPCR), while the detection of anti-*Leishmania* antibodies was performed using IFAT and ELISA. The exposure to *P. perniciosus* salivary antigens (SGH, rSP01 and rSP03B + rSP01) was measured by ELISA.

**Results:**

Ninety-seven (46.6%) and 116 (55.8%) of the 208 dogs were positive to *Leishmania* antibodies or DNA by at least one test at the beginning and end of the sand fly season, respectively. IFAT and ELISA presented a substantial agreement in the serodiagnosis of leishmaniosis. Discrepant PB nPCR results were obtained between sampling points. *Leishmania* DNA was detected in CS of 72 dogs at the end of the phlebotomine season. The presence of antibodies to the parasite measured by ELISA was significantly higher in dogs presenting clinical signs compatible with leishmaniosis at both sampling points. *Phlebotomus perniciosus* salivary antibodies were detected in 179 (86.1%) and 198 (95.2%) of the screened dogs at the beginning and end of the phlebotomine season, respectively.

**Conclusions:**

The association between ELISA positivity and clinical signs suggests its usefulness to confirm a clinical suspicion. CS nPCR seems to be an effective and non-invasive method for assessing early exposure to the parasite. PB nPCR should not be used as the sole diagnostic tool to monitor *Leishmania* infection. The correlation between the levels of antibodies to *P. perniciosus* saliva and *Leishmania* antibodies suggests the use of a humoral response to sand fly salivary antigens as biomarkers of *L. infantum* infection.
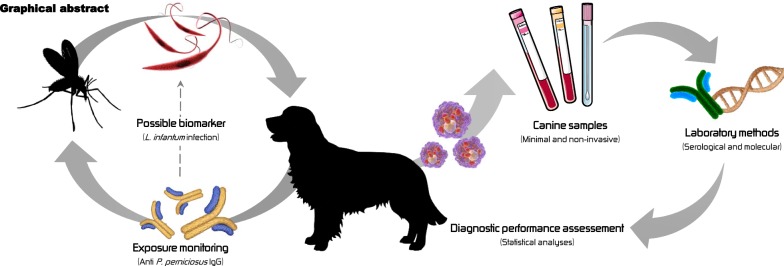

## Background

Canine leishmaniosis (CanL) caused by the protozoan *Leishmania infantum* is endemic in several countries of Central and South America, the Mediterranean Basin, Middle East and Asia [1]. Dogs are the main hosts and the principal reservoir hosts of human visceral infection. Parasites are transmitted by the bites of infected phlebotomine sand fly females (Diptera: Phlebotominae) with *Phlebotomus perniciosus* being the main vector in south-western Europe [2, 3].

The outcome of *L. infantum* infection is a consequence of intricate interactions between the protozoan and the genetic and immunological background of the host and ranges from the total absence of signs to severe systemic disease leading to death. In endemic areas, the percentage of subclinical infections is much more frequent than patent disease [4]. Despite the absence of clinical signs, subclinical dogs can serve as a source of infection for phlebotomine vectors [5]. Thus, apart from the confirmation of clinical suspicion in a single patient, the confirmation of *Leishmania* infection in dogs without clinical signs should be attempted to promote their monitoring through follow-up [6, 7].

The definitive diagnosis of CanL is complex and should integrate anamnesis, clinical, haematological and biochemical findings, as well as the detection of the parasite and/or the immune response developed by the host [8]. The commonly used laboratory techniques for the diagnosis include the direct detection of *Leishmania* DNA by molecular techniques such as polymerase chain reaction (PCR), and the indirect detection of antibodies against the parasite such as immunofluorescence antibody test (IFAT) or enzyme-linked immunosorbent assay (ELISA) [9]. The late appearance of specific antibodies along with the visceral tropism of the parasites makes sampling, as well as owner compliance, challenging as invasive collection of material biopsies, such as bone marrow, needs to be frequently repeated. Therefore, a diagnostic test using biological samples collected by minimal or non-invasive approaches is simpler to perform, better tolerated by animals and by far more acceptable to dog owners. The use of conjunctiva swabs as samples, coupled with a sensitive and specific PCR, has shown encouraging results for diagnosis, treatment follow-up and/or for assessing *Leishmania* exposure in dogs [10, 11]. Peripheral blood sampling allows the concomitant serological and molecular diagnosis of CanL. The main disadvantage of this biological sample is the inconsistency of parasitaemia over the course of infection, especially in subclinical animals, and therefore molecular tests applied on blood samples are mostly used as a complement of serological results [9].

During the blood meal, immunogenic components present in phlebotomine sand fly saliva are inoculated into the vertebrate host provoking the development of specific anti-saliva antibodies [12]. In endemic areas of leishmaniosis caused by *L. infantum*, the detection of these anti-sand fly salivary antibodies has proven to be a useful epidemiological biomarker for monitoring exposure of hosts to vectors and it might be used to estimate the risk for *Leishmania* infection. *Phlebotomus perniciosus*-specific salivary recombinant proteins have been produced, to overcome difficulties in obtaining appropriate amounts of whole salivary gland homogenates (SGH), especially in large scale epidemiological studies [13]. The evaluation of different recombinant antigens of *P. perniciosus* revealed yellow-related protein (rSP03B) and/or apyrase (rSP01) as the most promising candidates to replace SGH in the detection of *P. perniciosus* exposure in dogs [14–16] and other mammalian hosts [17].

The aim of this longitudinal study was to: (i) evaluate and compare diagnostic tests using minimally (i.e. peripheral blood) and non-invasive samples (i.e. conjunctiva cells) for monitoring *Leishmania*-dog infection; (ii) assess salivary antigens as markers of exposure to *P. perniciosus* and to *Leishmania* infection; and (iii) explore putative risks factors associated with *Leishmania* infection in sheltered dogs at the beginning, and the end of sand fly seasonal activity. The possible association between phlebotomine sand fly exposure and the presence of *Leishmania* infection was also investigated.

## Methods

### Animals

A total of 208 dogs (convenience sampling) from four private kennels from Lisbon and Setúbal districts belonging to the Metropolitan Lisbon region were enrolled in May and October 2011 (i.e. at the beginning and at the end of sand fly seasonal activity). CanL caused by *L. infantum* is endemic in both districts, and *Phlebotomus ariasi* and *P. perniciosus* are the proven vectors [18, 19]. *Phlebotomus sergenti* and *Sergentomyia minuta* are the other two sand fly species known to be endemic in the study area [2]. The four kennels have an elevated turnover due to new stray dog collections, adoptions and deaths. Whenever available, data on sex, breed, coat length, coat colour, age, use of insecticides and presence of clinical signs compatible with leishmaniosis (namely muscular atrophy, cutaneous lesions, epistaxis, lameness, lymphadenomegaly, onychogryphosis, ocular lesions, pale mucous membranes or weight loss; [20]) were recorded for each dog. Blood and serum samples were obtained from previous epidemiological studies regarding exposure to *L. infantum* or Toscana and sand fly fever Sicilian viruses [21–24].

### Samples

Peripheral blood (PB) (2–3 ml) was obtained by cephalic venipuncture from each animal and collected into serum-separating tubes and spotted on 3M filter paper. Serum was obtained by centrifugation and stored at − 20 °C until use in serological analyses and blood spotted on filter paper was preserved at 4 °C until DNA extraction.

Exfoliative epithelial cells were collected from the right and left conjunctiva (i.e. conjunctiva cells (CS)) of each animal using sterile cotton swabs. The swabs were rubbed against the surface of the lower eyelid, and then the cotton tip was immersed into a sterile 15 ml tube containing 1 ml of sterile saline. Twenty-four hours after incubation at 4 °C, swabs were pressed against the walls of the tube, and the saline containing eluted exfoliated cells was transferred into 2 ml sterile vials until DNA extraction [10]. Samples from each eyelid conjunctivas were processed separately.

### DNA extraction and PCR amplification

A commercial kit (Kit Citogene®; Citomed, Lisbons, Portugal) was used to extract DNA from blood on filter paper. Four discs of filter paper (4 mm in diameter each) were incubated with lysis buffer (150 μl) and 1.5 μl of proteinase K (20 mg/ml). Further DNA extraction followed the kit manufacturer’s instructions [24]. The saline samples containing eluted conjunctival cells were centrifuged at 3824×*g* for 10 min, and the pellets resuspended in 90 µl lysis buffer containing 10 µl of 2 mg/ml proteinase K. After 2 h incubation at 56 °C, the samples were incubated for 10 min at 95 °C and then centrifuged at 17,949×*g* for 10 min. DNA samples from both right and left conjunctivas were combined to increase DNA yield and stored at − 20 °C until used in the PCR assay [10].

Detection of *Leishmania* DNA was performed using a nested PCR (nPCR) protocol with primers targeting the small subunit ribosomal RNA (*SSU*-rDNA) gene [25]. A positive control containing genomic *L. infantum* DNA and a negative control without DNA template were included in each amplification. The DNA amplicons were resolved by conventional electrophoresis on 1.5% agarose gels stained with Green Safe Premium (Nzytech, Lisbon, Portugal), using a 100-bp DNA ladder as a molecular weight marker, then visualized under UV illumination. Positive samples yielded a predicted nPCR product of 358 bp.

### Detection of anti-*Leishmania* antibodies

Detection of anti-*Leishmania* antibodies was performed by an in-house IFAT and a commercial ELISA (Bordier Affinity Products SA, Crissier, Switzerland) as previously described [26]. Briefly, a *L. infantum* MON-1 (MCAN/PT/05/IMT-373) suspension of 10^7^ promastigotes was used as antigen, and anti-dog IgG (whole molecule)-FITC (Sigma-Aldrich, Missouri, USA) was used at a dilution of 1:20. A serum sample from a seropositive dog was used as positive control, while the serum sample of a dog from a non-endemic country of leishmaniosis (i.e. Czech Republic) and negative for *Leishmania* infection by both molecular and serological tests was used as negative control. The IFAT cut-off value was established at a serum dilution of 1:64. The ELISA was performed according to manufacturerʼs guidelines, and the result was considered positive when the absorbance of the analysed sample was higher than the absorbance of the weak positive control serum provided with the kit. The ELISA cut-off was 0.260, according to manufacturer’s instructions.

### Sand fly salivary proteins and detection of anti-*P. perniciosus* saliva antibodies

The colony of *Phlebotomus perniciosus* was maintained as previously described [27]. Salivary glands, dissected from 4–6 day-old females, were pooled in 20 mM Tris buffer with 150 mM NaCl then kept at − 80 °C until use. Recombinant salivary proteins from *P. perniciosus*, 35.5 kDa apyrase (rSP01) and 43 kDa yellow-related protein (rSP03B) were obtained from Apronex s.r.o. (Prague, Czech Republic), as previously described [13, 14].

Anti-*P. perniciosus* IgG was measured by ELISA as previously described [14]. Briefly, microtiter plates were coated either with salivary gland homogenate (SGH) (0.2 salivary gland per well) or with rSP03B (5 μg/ml) or with combination of two antigens rSP03B + rSP01 (5 μg/ml of each protein) in 20 mM carbonate-bicarbonate buffer (pH 9.5) and incubated overnight at 4 °C. The plates were washed with PBS + 0.05% Tween 20 (PBS-Tw) and incubated with blocking solution, 6% (w/v) low fat dry milk diluted in PBS-Tw at 37 °C for 60 min. Canine sera diluted 1:200 for SGH, and 1:100 for recombinant proteins in 2% (w/v) low fat dry milk/PBS-Tw were added to the wells (100 μl/well) after washing twice with PBS-Tw. After a 90 min incubation at 37 °C, the plates were washed with PBS-Tw and incubated at 37 °C for 45 min with the secondary antibody (anti-dog IgG (whole molecule)-FITC; Sigma-Aldrich) diluted 1:9,000 in PBS-Tw. The ELISA was developed using orthophenylenediamine (0.5 mg/ml) in a phosphate citrate buffer (pH 5.5) with 0.001% hydrogen peroxide (30%). Absorbance was measured at 492 nm using a NanoQuant plate reader (Infinite M200 Pro; Tecan, Männedorf, Switzerland). Each serum was tested in duplicate. Wells without serum (but coated with SGH) were used as blanks, while sera from six dogs living in a non-endemic country (Czech Republic) for leishmaniosis and without phlebotomine sand fly vectors, served as negative controls. The cut-off value was calculated by the addition of two standard deviations to the mean optical density (OD) of the control sera. The internal validity of ELISA assay was assured by including blanks and the same negative controls in each plate.

### Statistical analysis

Descriptive statistics and an exploratory data analysis were conducted for the main variables of the dataset. The normality of the quantitative variables was assessed by both Kolmogorov-Smirnov and Shapiro-Wilk tests. Associations between qualitative variables were explored through the Chi-square test, Fisher’s exact test or Freeman-Halton test. The cumulative incidence and relative risk (RR) factors for *Leishmania* infection (inferred by ELISA, IFAT, peripheral blood nPCR and conjunctival swab nPCR) were determined from a sample of dogs classified as not exposed (i.e. negative to all tests) at the beginning of the sand fly seasonal activity. RR values > 3 or < 0.33 were considered as potentially biologically important [28]. The Mann-Whitney-Wilcoxon test was used to compare the median levels of antibodies to *Leishmania* and *P. perniciosus* saliva between the beginning and end of the sand fly season. The agreement between the results of different tests performed to screen dogs for exposure to *Leishmania* and *P. perniciosus* saliva was estimated using Cohen’s kappa (*k*) test (*k* < 0, disagreement; *k* = 0, poor agreement; *k* > 0–0.20, slight agreement; *k* of 0.20–0.40, fair agreement; *k* of > 0.40–0.60, moderate agreement; *k* > 0.60–0.80, substantial agreement; *k* > 0.80–1.00 excellent agreement; [28]) and McNemar’s test used to test for significant differences between discordant results. Spearman’s correlation coefficient (*r*_*s*_) was determined to evaluate the strength (*r*_*s*_ = 0, none; *r*_*s*_ > 0–0.09, negligible; *r*_*s*_ ≥ 0.10–0.39, weak; *r*_*s*_ ≥ 0.40–0.69, moderate; *r*_*s*_ ≥ 0.70–0.90, strong; *r*_*s*_ > 0.90, very strong; [29]) of relationships between serum antibodies levels against *Leishmania* and against *P. perniciosus* saliva. Statistical significance was determined at *α* ≤ 0.05. The statistical analysis was conducted using IBM® SPSS® Statistics v 25.0, Epitools and GraphPad Prism v 6.01.

## Results

### *Leishmania-*positivity and anti-*P. perniciosus* antibodies at the beginning of sand fly seasonal activity

Ninety-seven (46.6%) of the 208 screened dogs were positive by at least one out of the four tests and were thus considered as having been exposed to *Leishmania* at the beginning of the sand fly seasonal activity, whereas the remaining 111 (53.4%) were negative to all tests and classified as not exposed (Table 1). Seventy-one (34.1%) dogs were positive by PB nPCR only and 4 (1.9%) by ELISA only; 22 (10.6%) dogs were positive to different combinations of techniques, but none were found to be positive by all (Table 2). Parasite DNA was detected in the peripheral blood of 85 (40.9%) dogs but in none of the conjunctival samples. Antibodies to *Leishmania* were detected in 26 (12.5%) sera by ELISA and in 14 (6.7%) sera by IFAT. ELISA OD values ranged between 0–1.680, while IFAT titres ranged between 0–2048 (Table 3). According to ELISA results, the presence of antibodies to the parasite was significantly higher in pure breed dogs in comparison to mongrels (*P* = 0.019), and in those dogs presenting clinical signs compatible with leishmaniosis (*P* = 0.035) (Table 2). No significant differences were detected in positivity to *Leishmania* by IFAT or PB nPCR among all the variables studied.

**Table 1:** Combined results of the serological and molecular analyses performed to assess the contact of kennelled dogs to *Leishmania* parasites at the beginning and end of sand fly season

Diagnostic test				Sand fly season (*n* = 208)	
ELISA	IFAT	Peripheral blood PCR	Conjunctival swab PCR^a^	Start, *n* (%)	End, *n* (%)
−	−	−	−	111 (53.4)	92 (44.2)
−	−	−	+	0 (0)	47 (22.6)
−	−	+	−	71 (34.1)	24 (11.5)
−	+	−	−	0 (0)	0 (0)
+	−	−	−	4 (1.9)	5 (2.4)
−	−	+	+	0 (0)	15 (7.2)
−	+	−	+	0 (0)	1 (0.5)
−	+	+	−	0 (0)	0 (0)
+	−	−	+	0 (0)	4 (1.9)
+	−	+	−	8 (3.8)	0 (0)
+	+	−	−	8 (3.8)	9 (4.3)
−	+	+	+	0 (0)	0 (0)
+	+	+	−	6 2.9)	6 (2.9)
+	+	−	+	0 (0)	4 (1.9)
+	−	+	+	0 (0)	1 (0.5)
+	+	+	+	0 (0)	0 (0)

^a^All negative at the beginning of the sand fly season

*Abbreviations*: ELISA, enzyme-linked immunosorbent assay; IFAT, immunofluorescence antibody test; PCR, polymerase chain reaction

**Table 2:** Contact of kennelled dogs to *Leishmania* parasites at the beginning and end of sand fly season—prevalence, cumulative incidence and relative risk factors by diagnostic test

Variable/category	Diagnostic test															
	ELISA				IFAT				Peripheral blood PCR				Conjunctival swab PCR			
	P_1_	P_2_	I	RR (95% CI)	P_1_	P_2_	I	RR (95% CI)	P_1_	P_2_	I	RR (95% CI)	P_1_	P_2_	I	RR (95% CI)
Sex, *n* (%)	*P* = 0.150	*P* = 0.091	*P* = 0.393		*P* = 0.200	*P* = 0.176	*P* = 1.000		*P* = 0.668	*P* = 0.342	*P* = 0.184			*P* = 0.473	*P* = 0.867	
Female^h^	12 (9.8)	13 (10.6)	2 (3.0)		6 (4.9)	9 (7.3)	1 (1.5)		52 (42.3)	30 (24.4)	19 (28.8)		0 (0)	45 (36.6)	21 (31.8)	
Male	14 (16.5)	16 (18.8)	3 (6.7)	2.20 (0.38–12.64)	8 (9.4)	11 (12.9)	1 (2.2)	1.47 (0.09–22.85)	33 (38.8)	16 (18.8)	8 (17.8)	0.62 (0.30–1.29)	0 (0)	27 (31.8)	15 (33.3)	1.05 (0.61–1.8)
Age group, *n* (%)^a^	*P* = 0.271	*P* = 0.709	*P* = 0.626	0.21 (0.02–2.97)	*P* = 0.769	*P* = 0.813	*P* = 0.474		*P* = 0.137	*P* = 1.000	*P* = 0.841			*P* = 0.831	*P* = 1.000	
< 1 year^h^	0 (0)	0 (0)	0 (0)		0 (0)	0 (0)	0 (0)		0 (0)	0 (0)	0 (0)		0 (0)	0 (0)	0 (0)	
1–7 years	13 (10.0)	17 (13.1)	3 (3.9)	Infinity	9 (6.9)	13 (10.0)	1 (1.3)	Infinity	47 (36.2)	26 (20.0)	17 (22.1)	Infinity	0 (0)	44 (33.8)	23 (29.9)	Infinity
> 7 years	11 (17.2)	10 (15.6)	2 (7.1)	Infinity	3 (4.7)	5 (7.8)	1 (3.6)	Infinity	31 (48.4)	13 (20.3)	7 (25.0)	Infinity	0 (0)	20 (31.3)	8 (28.6)	Infinity
Breed, *n* (%)	*P* = 0.018^*^	*P* = 0.318	*P* = 0.112		*P* = 0.089	*P* = 0.141	*P* = 0.202		*P* = 0.945	*P* = 0.146	*P* = 0.387			*P* = 0.318	*P* = 0.451	
Mongrel^h^	15 (9.4)^d^	19 (11.9)	3 (3.4)		8 (5.0)	12 (7.5)	1 (1.1)		65 (40.9)	31 (19.5)	19 (21.8)		0 (0)	51 (32.1)	26 (29.9)	
Crossed	3 (14.3)	4 (19.0)	2 (18.2)	5.27 (0.99–28.17)	2 (9.5)	3 (14.3)	1 (9.1)	7.91 (0.53–117.7)	8 (38.1)	5 (23.8)	3 (27.3)	1.25 (0.44–3.55)	0 (0)	10 (47.6)	4 (36.4)	1.22 (0.52–2.83)
Pure	8 (28.6)^d^	6 (21.4)	0 (0)	0	4 (14.3)	5 (17.9)	0 (0)	0	12 (42.9)	10 (35.7)	5 (38.5)	1.76 (0.80–3.90)	0 (0)	11 (39.3)	6 (46.2)	2.01 (0.62–6.57)
Coat length, *n* (%)^b^	*P* = 0.474	*P* = 0.804	*P* = 0.328		*P* = 0.765	*P* = 0.413	*P* = 0.105		*P* = 0.827	*P* = 0.508	*P* = 0.307			*P* = 0.975	*P* = 0.500	
Short^h^	15 (11.0)	18 (13.2)	2 (2.7)		8 (5.9)	11 (8.1)	0 (0)		55 (40.4)	28 (20.6)	16 (21.6)		0 (0)	47 (34.6)	22 (29.7)	
Medium or long	10 (14.5)	10 (14.5)	3 (8.3)	3.08 (0.54–17.64)	5 (7.2)	8 (11.6)	2 (5.6)	Infinity	29 (42.0)	17 (24.6)	11 (30.6)	1.41 (0.73–2.72)	0 (0)	24 (34.8)	13 (36.1)	1.22 (0.70–2.12)
Coat colour, *n* (%)^c^	*P* = 0.472	*P* = 0.861	*P* = 0.596		*P* = 0.636	*P* = 0.387	*P* = 0.305		*P* = 0.638	*P* = 0.015^*^	*P* = 0.195			*P* = 0.067	*P* = 0.035^*^	
Bright^h^	4 (8.0)	7 (14.0)	2 (6.7)		4 (8.0)	4 (8.0)	1 (3.3)		17 (34.0)	15 (30.0)^e^	9 (30.0)		0 (0)	23 (46.0)	15 (50.0)	
Mix	12 (12.6)	12 (12.6)	1 (2.0)	0.31 (0.03–3.23)	5 (5.3)	7 (7.4)	0 (0)	0	40 (42.1)	13 (13.7)^e,f^	8 (16.3)	0.54 (0.24–1.26)	0 (0)	33 (34.7)	15 (30.6)^g^	0.61 (0.35–1.06)
Dark	9 (15.8)	9 (15.8)	2 (6.5)	0.97 (0.15–6.43)	5 (8.8)	8 (14.0)	1 (3.2)	0.97 (0.06–14.78)	23 (40.4)	18 (31.6)^f^	10 (32.3)	1.07 (0.51–2.27)	0 (0)	14 (24.6)	6 (19.4)^g^	0.39 (0.17–0.86)
Clinical signs	*P* = 0.035*	*P* = 0.014*	*P* = 0.041*		*P* = 0.199	*P* = 0.126	*P* = 1.000		*P* = 0.236	*P* = 1.000	*P* = 0.677			*P* = 0.402	*P* = 1.000	
Absent^h^	15 (9.7)	22 (11.8)	3 (2.9)		8 (5.2)	16 (8.6)	2 (1.9)		67 (43.2)	42 (22.5)	26 (25.2)		0 (0)	63 (33.7)	34 (33.0)	
Present	11 (20.8)	7 (33.3)	2 (25.0)	8.58 (1.67–44.17)	6 (11.3)	4 (19.0)	0 (0)	0	18 (34.0)	4 (19.0)	1 (12.5)	0.50 (0.08–3.19)	0 (0)	9 (42.9)	2 (25.0)	0.76 (0.22–2.6)
Insecticides	*P* = 0.701	*P* = 0.068	*P* = 0.257		*P* = 0.605	*P* = 0.068	*P* = 0.359		*P* = 0.501	*P* = 0.021^*^	*P* = 0.010^*^			*P* = 0.852	*P* = 0.945	
No^h^	23 (12.2)	20 (11.8)	3 (3.4)		12 (6.4)	13 (7.7)	1 (1.1)		78 (41.5)	32 (18.9)	17 (19.1)		0 (0)	58 (34.3)	29 (32.6)	
Yes	1 (5.6)	9 (23.1)	2 (9.1)	2.7 (0.48–15.17)	0 (0.0)	7 (17.9)	1 (4.5)	4.04 (0.26–62.17)	6 (33.3)	14 (35.9)	10 (45.5)	2.38 (1.27–4.45)	0 (0)	14 (35.9)	7 (31.8)	0.98 (0.49–1.93)
Ac anti-rSP03B, *n* (%)	*P* = 0.705	*P* = 1.000	*P* = 0.548		*P* = 0.453	*P* = 1.000	*P* = 0.269		*P* = 0.213	*P* = 0.038^*^	*P* = 0.063			*P* = 0.566	*P* = 0.206	
No^h^	9 (11.4)	4 (13.3)	1 (6.3)		4 (5.1)	3 (10.0)	1 (6.3)		28 (35.4)	11 (36.7)	7 (43.8)		0 (0)	9 (30.0)	3 (18.8)	
Yes	17 (13.2)	25 (14.0)	4 (4.2)	0.67 (0.08–5.65)	10 (7.8)	17 (9.6)	1 (1.1)	0.17 (0.01–2.56)	57 (44.2)	35 (19.7)	20 (21.1)	0.48 (0.24–0.95)	0 (0)	63 (35.4)	33 (34.7)	1.85 (0.64–5.33)
Ac anti-rSP03B + rSP01, *n* (%)	*P* = 0.265	*P* = 0.288	*P* = 0.282		*P* = 0.335	*P* = 0.382	*P* = 1.000		*P* = 0.733	*P* = 0.556	*P* = 0.358			*P* = 0.051	*P* = 0.680	
No^h^	2 (5.9)	4 (22.2)	1 (14.3)		1 (2.9)	1 (5.6)	0 (0)		13 (38.2)	5 (27.8)	3 (42.9)		0 (0)	10 (55.6)	3 (42.9)	
Yes	24 (13.8)	25 (13.2)	4 (3.8)	0.27 (0.04–2.10)	13 (7.5)	19 (10.0)	2 (1.9)	Infinity	72 (41.4)	41 (21.6)	24 (23.1)	0.54 (0.21–1.36)	0 (0)	62 (32.6)	33 (31.7)	0.74 (0.30–1.82)
Ac anti-SGH, *n* (%)	*P* = 0.207	*P* = 1.000	*P* = 0.442		*P* = 1.000	*P* = 0.479	*P* = 1.000		*P* = 0.067	*P* = 0.256	*P* = 0.352			*P* = 0.378	*P* = 0.521	
No^h^	4 (7.5)	3 (11.5)	1 (8.3)		3 (5.7)	1 (3.8)	0 (0)		16 (30.2)	8 (30.8)	4 (33.3)		0 (0)	11 (42.3)	5 (41.7)	
Yes	22 (14.2)	26 (14.3)	4 (4.0)	0.48 (0.06–3.99)	11 (7.1)	19 (10.4)	2 (2.0)	Infinity	69 (44.5)	38 (20.9)	23 (23.2)	0.7 (0.29–1.68)	0 (0)	61 (33.5)	31 (31.3)	0.75 (0.36–1.56)
Total, *n* (%)	26 (12.5)	29 (13.9)	5 (4.5)		14 (6.7)	20 (9.6)	2 (1.8)		85 (40.9)	46 (22.1)	27 (24.3)		0 (0)	72 (34.6)	36 (32.4)	

^a^P_1_ and P_2_ (*n* = 195); I (*n* = 106)

^b^P_1_ and P_2_ (*n* = 205); I (*n* = 110)

^c^P_1_ and P_2_ (*n* = 202); I (*n* = 110)

^d^*P* = 0.019

^e^*P* = 0.018

^f^*P* = 0.008

^g^*P* = 0.012

^h^Reference category

^*^Significant statistical association (α ≤ 0.05)

*Abbreviations*: ELISA, enzyme-linked immunosorbent assay; IFAT, immunofluorescence antibody test; PCR, polymerase chain reaction; P_1_, prevalence of *Leishmania* infection at the beginning of the sand fly season (*n* = 208); P_2_, prevalence of *Leishmania* infection at the end of the sand fly season (*n* = 208); I, cumulative incidence (*n* = 111); RR, relative risk; 95% CI, 95% confidence interval; rSP03B, 43 KDa yellow-related protein; rSP03B + rSP01, rSP03B and 35.5 kDa apyrase; SGH, salivary gland homogenate

**Table 3:** Comparison of median levels of antibodies to *Leishmania* or *Phlebotomus perniciosus* at the beginning and end of the sand fly season

Serological technique	Sand fly season (*n* = 208)				*P*-value
	Start		End		
	Median (IQR)	Range	Median (IQR)	Range	
*P. perniciosus*					
ELISA-rSP03B	0.350 (0.282–0.496)	0.117–1.406	0.369 (0.280–0.491)	0.143–1.066	0.793
ELISA-rSP03B + rSP01	0.370 (0.292–0.508)	0.129–1.253	0.393 (0.308–0.556)	0.164–1.451	0.060
ELISA-SGH	0.237 (0.186–0.343)	0.083–1.444	0.282 (0.232–0.380)	0.110–1.404	< 0.001
*Leishmania*					
ELISA	0.037 (0.024–0.064)	0.000–1.680	0.038 (0.025–0.071)	0–1.620	0.457
IFAT	0 (0–0)	0–2048	0 (0–0)	0–9192	0.919

*Abbreviations*: ELISA, enzyme-linked immunosorbent assay; IFAT, immunofluorescence antibody test; rSP03B, 43 KDa yellow-related protein; rSP03B + rSP01, rSP03B and 35.5 kDa apyrase; SGH, salivary gland homogenate; IQR, interquartile interval (quartile 1–quartile 3)

One hundred seventy-nine (86.1%) dogs were seropositive to *P. perniciosus* saliva (Table 4). Antibodies to rSP03B + rSP01, SGH and rSP03B were detected in 174 (83.7%), 155 (74.5%) and 129 (62.0%) sera, respectively. One hundred twenty-one (58.2%) dogs were positive to the three salivary antigens, while 58 (27.9%) dogs were positive to different combinations of them. The OD values and frequency distribution for each *P. perniciosus* salivary antigen are summarized in Table 3.

**Table 4:** Combined results of the serological analyses performed to assess the exposure of kennelled dogs to *Phlebotomus perniciosus* at the beginning and end of the sand fly season

Salivary gland antigen			Sand fly season (*n* = 208)	
rSP03B	rSP03B + rSP01	SGH	Start, *n* (%)	End, *n* (%)
−	−	−	29 (13.9)	10 (4.8)
−	−	+	5 (2.4)	5 (2.4)
−	+	−	16 (7.7)	6 (2.9)
+	−	−	0 (0)	2 (1.0)
−	+	+	29 (13.9)	9 (4.3)
+	−	+	0 (0)	1 (0.5)
+	+	−	8 (3.8)	8 (3.8)
+	+	+	121 (58.2)	167 (80.3)

*Abbreviations*: rSP03B, 43 KDa yellow-related protein; rSP03B + rSP01, rSP03B and 35.5 kDa apyrase; SGH, salivary gland homogenate

A substantial agreement was found between ELISA and IFAT for the detection of antibodies against the parasite (*k* = 0.671) and a moderate agreement was obtained between the three salivary antigens (*k* = 0.484 for rSP03B and rSP03B + rSP01; *k* = 0.542 for rSP03B and SGH; *k* = 0.584 for rSP03B + rSP01 and SGH) (Table 5). Discordant results were found between the different serological techniques for the detection of *Leishmania-* and *P. perniciosus-*specific antibodies and PB nPCR.

**Table 5:** Agreement rates between diagnostic tests results at the beginning and end of the sand fly season

Comparison	Sand fly season											
	Start						End					
	*P*-value	Kappa	95% CI	PA (%)	NA (%)	OA (%)	*P*-value	Kappa	95% CI	PA (%)	NA (%)	OA (%)
A *vs* B	0.002	0.671	0.501–0.842	70.0	98.8	94.2	0.016	0.747	0.605–0.888	77.6	97.0	94.7
A *vs* C	< 0.001	0.075	− 0.031–0.181	25.2	72.8	60.1	0.041	0.019	− 0.115–0.153	18.7	82.1	70.7
A *vs* D^a^							< 0.001	− 0.026	− 0.138–0.087	17.8	73.7	60.1
A *vs* E	< 0.001	0.014	− 0.059–0.009	21.9	53.6	41.2	< 0.001	0.002	− 0.041–0.045	24.2	24.9	24.5
A *vs* F	< 0.001	0.029	− 0.007–0.064	24.0	29.6	26.9	< 0.001	− 0.018	− 0.058–0.022	22.8	14.2	18.8
A *vs* G	< 0.001	0.037	− 0.014–0.088	24.3	41.7	34.1	< 0.001	0.008	− 0.030–0.046	35.3	24.6	23.6
B *vs* C	< 0.001	0.006	− 0.074–0.089	12.2	72.6	58.2	< 0.001	0.055	− 0.078–0.188	18.2	84.6	74.0
B *vs* D^a^							< 0.001	− 0.049	− 0.144–0.046	10.9	74.7	60.6
B *vs* E	< 0.001	0.021	− 0.031–0.073	14.0	55.0	40.9	< 0.001	− 0.001	− 0.038–0.035	17.2	24.8	21.2
B *vs* F	< 0.001	0.016	− 0.009–0.040	13.8	29.0	22.1	< 0.001	0.008	− 0.014–0.030	18.1	16.5	17.3
B *vs* G	< 0.001	0.008	− 0.032–0.047	13.0	40.5	29.3	< 0.001	0.018	− 0.007–0.043	18.8	23.4	21.2
C *vs* D^a^							0.007	0.002	− 0.132–0.132	27.1	71.1	58.7
C *vs* E	< 0.001	0.079	− 0.044–0.202	53.3	50.5	51.9	< 0.001	− 0.060	− 0.128–0.008	31.3	19.8	26.0
C *vs* F	< 0.001	0.015	− 0.072–0.103	55.6	26.8	44.7	< 0.001	− 0.013	− 0.060–0.034	34.8	14.4	26.0
C *vs* G	< 0.001	0.010	− 0.004–0.204	57.5	42.1	51.0	< 0.001	− 0.031	− 0.089–0.028	33.3	19.2	26.9
D *vs* E^a^							< 0.001	0.022	− 0.051–0.095	50.4	25.3	40.4
D *vs* F^a^							< 0.001	− 0.058	− 0.123–0.008	47.3	10.4	33.7
D *vs* G^a^							< 0.001	− 0.031	− 0.104–0.041	48.0	18.5	36.5
E *vs* F	< 0.001	0.484	0.369–0.598	85.2	60.2	78.4	0.010	0.580	0.408–0.752	95.1	62.5	91.4
E *vs* G	< 0.001	0.542	0.425–0.660	85.2	68.2	79.8	0.540	0.505	0.334–0.677	93.3	54.1	88.5
F *vs* G	< 0.001	0.584	0.451–0.716	91.2	66.7	86.1	0.118	0.494	0.304–0.684	94.6	54.6	90.4

^a^*Leishmania* DNA was not detected at the beginning of the phlebotomine sand fly season by conjunctival swab PCR

*Abbreviations*: A, enzyme-linked immunosorbent assay; B, immunofluorescence antibody test; C, peripheral blood-polymerase chain reaction (PCR); D, conjunctival swab-PCR; E, 43 KDa yellow-related protein; F, 43 kDa yellow-related protein and 35.5 kDa apyrase; G, salivary gland homogenate; PA, positive agreement; NA, negative agreement; OA, overall agreement

Significant positive correlations between the antibody levels to the three salivary antigens were observed (*r*_*s*_ = 0.928, *P* < 0.001, between rSP03B + rSP01 and rSP03B; *r*_*s*_ = 0.841, *P* < 0.001, between rSP03B + rSP01 and SGH; *r*_*s*_ = 0.820, *P* < 0.001, between rSP03B and SGH) (Additional file 1: Figure S1). The OD of the antibody levels to the three salivary antigens and to both rSP03B + rSP01/SGH were found to be positively correlated with the ELISA OD (rSP03B: *r*_*s*_ = 0.244, *P* < 0.001; rSP03B + rSP01: *r*_*s*_ = 0.286, *P* < 0.001; SGH: *r*_*s*_ = 0.250, *P* < 0.001) and IFAT titres (rSP03B + rSP01: *r*_*s*_ = 0.146, *P* < 0.035; SGH: *r*_*s*_ = 0.178, *P* < 0.010) to *L. infantum*, respectively. The increase in ELISA OD levels of antibodies to *Leishmania* was also significantly correlated with the increase in IFAT titres (*r*_*s*_ = 0.473, *P* < 0.001).

### *Leishmania-*positivity and anti-*P. perniciosus* antibodies at the end of sand fly seasonal activity

At the end of sand fly seasonal activity 116 (55.8%) of the 208 screened dogs were positive by at least one test, while 92 (44.2%) remained negative (Table 1). Forty-seven (22.6%) dogs were positive by CS nPCR only, 24 (11.5%) by PB nPCR only, and 5 (2.4%) by ELISA only; 39 (18.8%) dogs were positive to different combinations of techniques, but none were found to be positive by all. Parasite DNA was detected in the peripheral blood and conjunctival samples of 46 (22.1%) and 72 (34.6%) dogs, respectively. The PB nPCR result was found significantly associated with coat colour (*P* = 0.015), insecticide treatment (*P* = 0.021) and antibodies to rSP03B salivary antigen (*P* = 0.038) (Table 3). The presence of *Leishmania* DNA was significantly higher in dogs with light (*P* = 0.018) or dark (*P* = 0.008) coat in comparison to those with mix colour coat, in dogs treated with insecticides (*P* = 0.021) and in those lacking specific antibodies to rSP03B (*P* = 0.038). Dogs without significant levels of antibodies to this salivary antigen had a RR 0.48 times lower (95% CI: 0.24–0.95) to be found positive by PB nPCR. The incidence of the detection of parasite DNA in the peripheral blood and conjunctival cells was significantly higher in dogs treated with insecticides (*P* = 0.010, RR = 2.38, 95% CI: 1.25–4.45) and with coloured coat (*P* = 0.035), respectively. Further, dogs with dark coat had a RR 0.39 times lower (95% CI: 0.17–0.86) to be found positive by CS nPCR than dogs with light coat.

Antibodies to *Leishmania* were detected in 29 (13.9%) sera by ELISA and in 20 (9.6%) sera by IFAT. The ELISA result was significantly associated with clinical signs (*P* = 0.014). Dogs presenting clinical signs compatible with leishmaniosis had a RR 8.58 times higher (95% CI: 1.67–44.17) to be seropositive by ELISA than those without clinical manifestations. Seroreversion in ELISA and IFAT occurred in 15.4% (4/26) and 7.1 % (1/14) dog sera, respectively. Further, seroconversion was approximately 3% in both techniques: ELISA: 3.8% (7/182); IFAT: 3.1% (6/194).

One hundred ninety-eight (95.2%) dogs were seropositive to *P. perniciosus* saliva (Table 4). Antibodies to rSP03B + rSP01, SGH and rSP03B were detected in 190 (91.3%), 182 (87.5%) and 178 (85.6%), respectively. One hundred sixty-seven (80.3%) dogs were positive to the three salivary antigens, while 31 dogs were positive to different combinations of them. The OD values and frequency distribution for each *P. perniciosus* salivary antigen are summarized in Table 2.

A substantial agreement was found between ELISA and IFAT for the detection of antibodies against the parasite (*k* = 0.747) and a moderate agreement was obtained between the three salivary antigens (*k* = 0.580 for rSP03B and rSP03B + rSP01; *k* = 0.505 for rSP03B and SGH; *k* = 0.494 for rSP03B + rSP01 and SGH) (Table 5). Discordant results were found between the different serological techniques for the detection of *Leishmania*- and *P. perniciosus-*specific antibodies, and for the molecular detection of parasite DNA.

Significant positive correlations between the antibody levels to the three salivary antigens were observed (between rSP03B + rSP01 and rSP03B: *r*_*s*_ = 0.932, *P* < 0.001; between rSP03B + rSP01 and SGH: *r*_*s*_ = 0.730, *P* < 0.001; between rSP03B and SGH: *r*_*s*_ = 0.719, *P* < 0.001) (Additional file 2: Figure S2). A significant positive correlation between OD values to the three salivary antigens and ELISA OD (rSP03B + rSP01: *r*_*s*_ = 0.140, *P* < 0.140; rSP03B: *r*_*s*_ = 0.153, *P* < 0.028; SGH: *r*_*s*_ = 0.232, *P* = 0.001) to *L. infantum* was also found. The increase in ELISA OD levels of antibodies to *Leishmania* was significantly correlated with the increase in IFAT titre (*r*_*s*_ = 0.584, *P* < 0.001).

## Discussion

In endemic areas of zoonotic leishmaniosis caused by *L. infantum*, both clinical and subclinical dogs represent a source of parasites to the vectors, contributing to the maintenance of the endemicity of the disease [5]. Therefore, early detection of *Leishmania* infection in both groups of infected dogs (i.e. with and without clinical signs) is essential to control the dissemination of the parasite to other dogs and humans [7, 30].

Despite clinical staging of CanL is still a matter of debate [8, 31, 32], the diagnosis of disease in clinical dogs from endemic areas is easier to achieve than the assessment of *L. infantum* infection in subclinical dogs [33, 34]. The confirmation of cryptic infections is difficult due to the absence of gold standards for their diagnosis, and due to the lack of compliance of owners to screen the presence of the parasite in their dogs, considered by them as “healthy”, especially when sampling is invasive and needs to be frequently repeated [9, 10, 30].

The most useful diagnostic approaches for the investigation of infection include the detection of specific serum anti-leishmanial antibodies and parasite DNA in tissues by molecular techniques [9]. Thus, in the present study, we evaluated the molecular and serological performance of blood, a minimally invasive biological sample, to detect the presence of *Leishmania* antibodies or its DNA in sheltered dogs at the beginning and end of the sand fly activity period. Antibodies to *Leishmania* were detected in 26 (12.5%) and 29 (13.9%) sera by ELISA, and in 14 (6.7%) and in 20 (9.6%) sera by IFAT, at the beginning and the end of the sand fly season, respectively, reinforcing the endemicity of the parasitosis in Metropolitan region of Lisbon [15, 23]. The increased number of seropositive dogs detected by both techniques at the end of the study might reflect on one hand, an infection that was not detectable at the start of the study, due to the low production of antibodies at the beginning of *Leishmania* infection [35]; this hypothesis is highly probable as most of the sheltered dogs were previously stray living in deficient health and nutritional conditions, and representing, therefore, an easier target for phlebotomine sand fly biting and for infection. On the other hand, the increased number of seropositive dogs at the end of sand fly season might represent an infection that occurred in the kennel, as the seroconversion rate by ELISA and IFAT was 4.5% and 1.8%, respectively, based on incidence values. Both techniques have been shown to have a high sensitivity as they were able to detect canine antibodies to the parasite between one to five months after experimental infection of dogs with *L. infantum* [26]. Further, *L. infantum* vectors were collected in the same year of the study in the screened area [2], and thus dogs could have been bitten by infected sand flies. Despite the overall increase in the number of dogs considered seropositive at the end of the sand fly season, some animals that were positive at the beginning of the study seroreverted. Seroreversion based on different diagnostic techniques over time has already been reported among naturally infected dogs [36] which may reflect exposure to the parasite followed by its clearance [37].

Both serological techniques presented a substantial agreement and positive correlation at both sampling points supporting that they can equally be used in the serological diagnosis of this parasitosis; also, and as both are quantitative, they can be used to follow-up antibody production and response to treatment [9, 31]. The presence of antibodies to *Leishmania* detected by ELISA was significantly higher in pure breed dogs in comparison to mongrels, corroborating that the latter seem to have developed a certain resistance to *Leishmania* infection [38]. More importantly, an association between ELISA positivity and clinical signs was observed, with dogs presenting clinical signs compatible with leishmaniosis having a RR 8.58 times higher to be seropositive by this technique than those without clinical manifestations, supporting its use to confirm a clinical suspicion. In fact, in a previous study on experimentally infected dogs, the association between clinical signs and seropositivity to this commercial ELISA was suggested as a predictive marker of the development of infection to disease [26].

Dogs not presenting antibodies to *P. perniciosus* rSP03B protein had a RR 0.48 times lower to be found positive by PB nPCR than those having this anti-sand fly saliva antigen, suggesting that the lack of canine antibodies to this specific salivary protein reflects the lower probability to encounter infected bites.

The incidence of infection measured by PB nPCR was 24.3%, and it was significantly higher in dogs treated with insecticides. Despite the use of topical insecticides, which has been shown to be effective in reducing the incidence of *Leishmania* infection in dogs [39], the significantly higher probability of dogs treated with ectoparasiticides to harbour parasite DNA in the peripheral blood is not entirely surprising, as neither the compliance of application, nor the correct administration and effectivity of insecticides were evaluated. Whether the obtained incidence was due to transient or to real infection could not be verified, as the 27 dogs that became positive by PB nPCR at the end of the sand fly season were not followed-up further. Nevertheless, and due to the discrepancy of positive PB nPCR results at the beginning and end of sand fly seasonal activity (Table 2), together with the insufficient data regarding the duration and consistency of parasitaemia over the course of infection [10, 11, 26, 40], DNA-positivity in PB should not be used as sole diagnostic tools for disease diagnosis.

The diagnostic performance of conjunctiva cells obtained non-invasively, coupled with nPCR to detect the presence of *Leishmania* DNA was also evaluated, yielding positive results only at the end of phlebotomine seasonal activity. Out of the 72 dogs harbouring parasite DNA, 47 were positive by CS nPCR only; this situation has been reported previously in naturally and experimentally infected dogs and related to a recent parasite contact [11, 40, 41]. Whether the contact with the parasite would evolve to the clearance or establishment of infection could not be analysed as dogs were not followed-up after October. Nevertheless, CS nPCR seems to be effective for assessing early exposure to the parasite. An association between the incidence of the detection of parasite DNA in conjunctival cells and colour of coat was observed, with dogs presenting dark coat having a RR 0.39 times lower by this technique than those with light coat. The attractiveness of phlebotomine sand flies to different light colours of CDCs traps has been demonstrated [42]; however, it is currently unknown whether the eyes of *L. infantum* vectors react more to light coat than to dark.

Overall, the discordance found between the serological and molecular techniques for determining *Leishmania* infection/exposure and incidence confirms that they do not have the same diagnostic performance and reinforces that *Leishmania*-dog contact should be monitored using more than one technique [9, 11, 34]. The early detection of parasite contact will allow dogs to be followed-up to confirm the clearance or establishment of infection, and to adopt control measures to avoid parasite transmission to the vectors.

The quantification of anti-*P. perniciosus* saliva antibodies in vertebrate hosts of *L. infantum* has proven to be an effective way of measuring exposure to this parasite vector [14–17, 43, 44]. As in the year that the study was conducted, the sand fly activity in the Metropolitan region of Lisbon started in May and ended in October [2], it is not surprisingly the high overall levels of seropositivity to anti-sand fly saliva antigens at the beginning (86.1%) and at the end (95.2%) of sand fly season, confirming the CanL endemicity status for the region [24]. As antibodies to sand fly saliva decay after the end of the biting season [14], the detection of antibodies to *P. perniciosus* saliva in a high percentage of dogs at the beginning of the season was probably related to their re-exposure to sand flies following antigenic priming in the previous season [15]. On the other hand, and since antibodies against saliva rise during summer months when sand fly abundance is higher [14], the increased number of dogs seropositive to salivary antigens at the end of the sand fly season was probably related with the exposure to more sand fly bites, as a second peak of *P. perniciosus* activity in Portugal was observed in September [2].

Antibodies recognizing SGH, rSP03B and rSP03B + rSP01 followed similar dynamics throughout the study reinforcing their use to assess exposure to *P. perniciosus* in dogs living in *L. infantum* endemic areas. A combination of the two recombinant proteins (rSP03B + rSP01) showed a better performance and higher mean OD values than the single rSP03B to detected dogs exposure to *P. perniciosus*. Similar results were previously published [13], while other studies on *P. perniciosus* prefer to use a single antigen rSP03B [14–16]. In *Lutzomyia longipalpis*, the main vector of *L. infantum* in the New World, a combination of two recombinant salivary proteins were successfully used [45].

In the study area, *P. perniciosus* is sympatric with *P. ariasi* [2], a closely related species of the subgenus *Larroussius*; thus, the high percentage of detection of antibodies to SGH might reflect cross-reactivity with antibodies against *P. ariasi* [12, 16]. As the presence and abundance of sand fly species responsible for *L. infantum* transmission varies according to the location, and throughout the transmission season, the sole use of SGH to specifically measure exposure to *P. perniciosus*, the most abundant *L. infantum* vector in the Western Mediterranean, can be hampered; however, this cross-reactivity might be an advantage, as both *Larroussius* species are proven *L. infantum* vectors [3].

The use of antibodies to sand fly salivary antigens as risk markers of *L. infantum* infection in dogs has been repeatedly evaluated but remains controversial: positive [14–16], negative [43] or no correlations [15] between the levels of anti-*P. perniciosus* saliva and *L. infantum* infection have been reported in dogs from endemic areas of leishmaniosis. As the antigenic response to phlebotomine sand fly saliva reflects sand fly bites, whether the sand fly is infected or not, while *Leishmania* infection only occurs if the vertebrate host after being inoculated with the parasite cannot clear it, the relationship between the dynamics of antibodies against sand fly saliva and a subsequent *Leishmania* infection can only be evaluated in longitudinal studies. In the present study, a significant positive (although low) correlation between OD values to the three salivary antigens and ELISA OD to *L. infantum* was found in both time intervals and between IFAT titres and OD values to salivary antigens at the beginning of the phlebotomine season, reinforcing their potential usefulness as biomarkers of *L. infantum* infection [15, 16, 46].

## Conclusions

The association between ELISA positivity and clinical signs compatible with leishmaniosis suggests its use to confirm a clinical suspicion. CS nPCR seems to be an effective and non-invasive method for assessing early exposure to the parasite. Salivary antigens are useful to monitor dog exposure to *P. perniciosus* bites in areas where this sand fly species is present. The correlation between the levels of antibodies to *P. perniciosus* saliva and *Leishmania* antibodies suggests the use of canine humoral response to salivary antigens as biomarkers of *L. infantum* infection.

## Supplementary information

**Additional file 1: Figure S1.** Correlations between serum antibodies levels at the beginning of sand fly season. **a** Between 43 KDa yellow-related protein (rSP03B) and rSP03B + 35.5 kDa apyrase (rSP03B + rSP01). **b** Between rSP03B and salivary gland homogenate (SGH). **c** Between rSP03B and *Leishmania* [enzyme-linked immunosorbent assay (ELISA)]. **d** Between rSP03B and *Leishmania* immunofluorescence antibody test (IFAT). **e** Between rSP03B + rSP01and SGH. **f** Between rSP03B + rSP01 and *Leishmania*-ELISA. **g** Between rSP03B + rSP01 and *Leishmania*-IFAT. **h** Between SGH and *Leishmania*-ELISA. **i** Between SGH and *Leishmania*-IFAT. **j** Between *Leishmania*-ELISA and *Leishmania*-IFAT. *Abbreviation*: OD, optical density.

**Additional file 2: Figure S2.** Correlations between serum antibodies levels at the end of sand fly season. **a** Between 43 KDa yellow-related protein (rSP03B) and rSP03B + 35.5 kDa apyrase (rSP03B + rSP01). **b** Between rSP03B and salivary gland homogenate (SGH). **c** Between rSP03B and *Leishmania* [enzyme-linked immunosorbent assay (ELISA)]. **d** Between rSP03B and *Leishmania* immunofluorescence antibody test (IFAT). **e** Between rSP03B + rSP01and SGH. **f** between rSP03B + rSP01 and *Leishmania*-ELISA. **g** Between rSP03B + rSP01 and *Leishmania*-IFAT. **h** Between SGH and *Leishmania*-ELISA. **i** Between SGH and *Leishmania*-IFAT. **j** Between *Leishmania*-ELISA and *Leishmania*-IFAT. *Abbreviation*: OD, optical density.

## Data Availability

The data supporting the conclusions of this article are included within the article and its Additional files.

## Abbreviations

CanL: canine leishmaniosis
CI: confidence interval
CS: conjunctival cells
ELISA: enzyme-linked immunosorbent assay
IFAT: immunofluorescence antibody test
nPCR: nested PCR
OD: optical density
PB: peripheral blood
PCR: polymerase chain reaction
RR: relative risk
rSP01: recombinant apyrase
rSP03B: recombinant yellow-related protein
SGH: salivary gland homogenate
