# Supplementary figures and images for: Monitoring Leishmania infection and exposure to Phlebotomus perniciosus using minimal and non-invasive canine samples
Source: Parasit Vectors. 2020 Apr 21;13:119. doi: 10.1186/s13071-020-3993-7 (PMC7171869; doi:10.1186/s13071-020-3993-7)

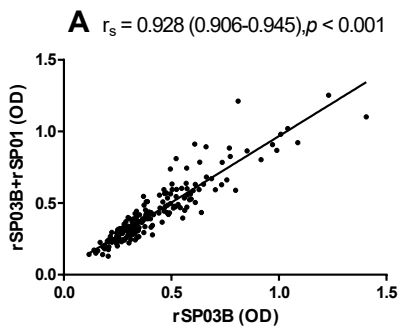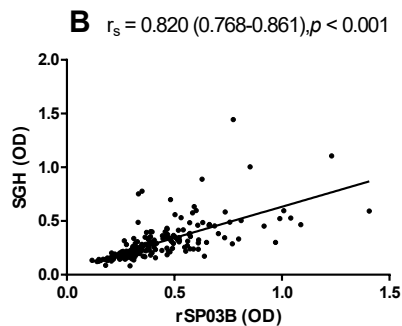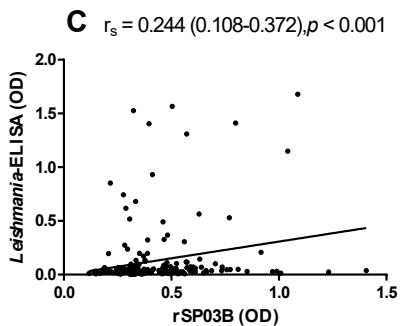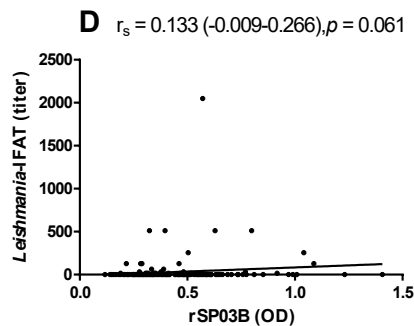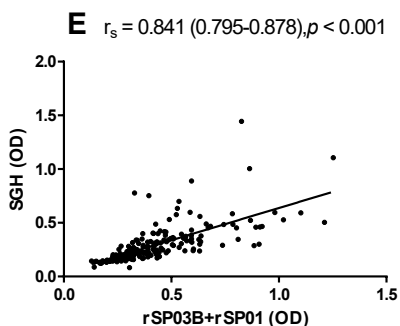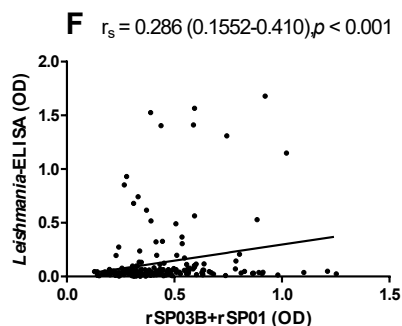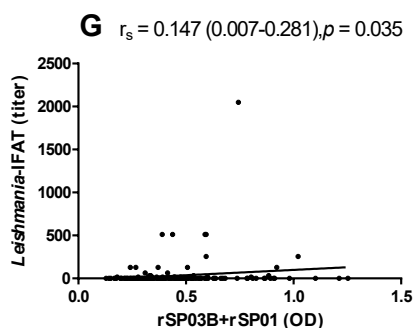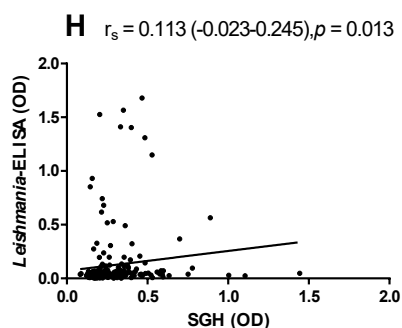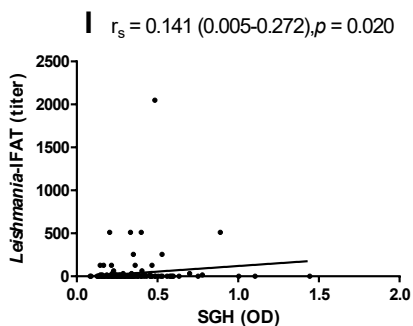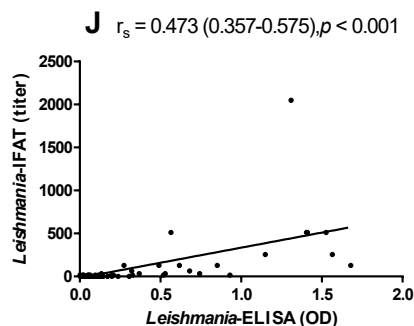

Supplement: Supplementary file 1 — Additional file 1: Figure S1. Correlations between serum antibodies levels at the beginning of sand fly season. a Between 43 KDa yellow-related protein (rSP03B) and rSP03B + 35.5 kDa apyrase (rSP03B + rSP01). b Between rSP03B and salivary gland homogenate (SGH). c Between rSP03B and Leishmania [enzyme-linked immunosorbent assay (ELISA)]. d Between rSP03B and Leishmania immunofluorescence antibody test (IFAT). e Between rSP03B + rSP01and SGH. f Between rSP03B + rSP01 and Leishmania-ELISA. g Between rSP03B + rSP01 and Leishmania-IFAT. h Between SGH and Leishmania-ELISA. i Between SGH and Leishmania-IFAT. j Between Leishmania-ELISA and Leishmania-IFAT. Abbreviation: OD, optical density. [file 13071_2020_3993_MOESM1_ESM.pdf]

**A**  $r_s = 0.932 (0.911-0.948), p < 0.001$

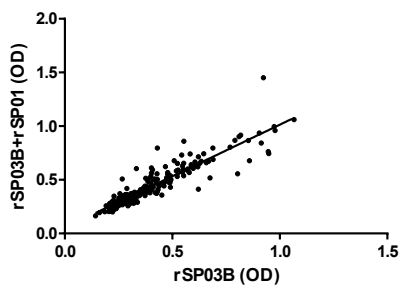

**B**  $r_s = 0.719 (0.644-0.780), p < 0.001$

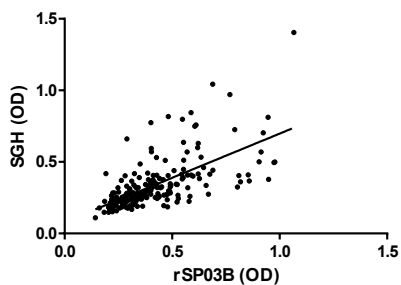

**C**  $r_s = 0.153 (0.013-0.287), p = 0.028$

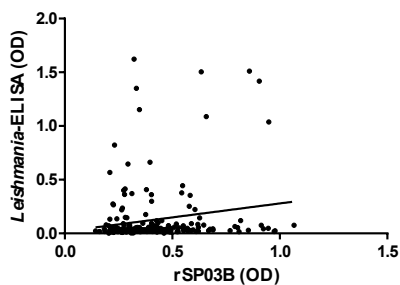

**D**  $r_s = 0.087 (-0.054-0.224), p = 0.214$

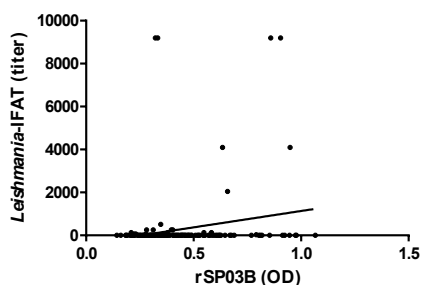

**E**  $r_s = 0.730 (0.658-0.790), p < 0.001$

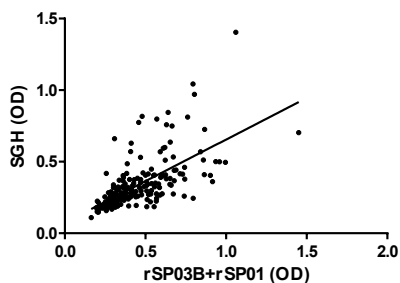

**F**  $r_s = 0.140 (-0.00-0.275), p = 0.044$

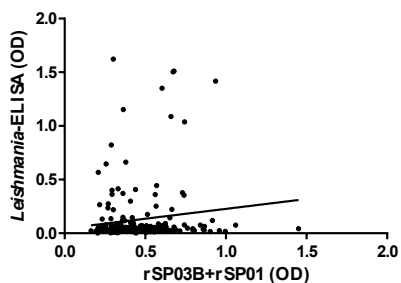

**G**  $r_s = 0.104 (-0.036-0.240), p = 0.135$

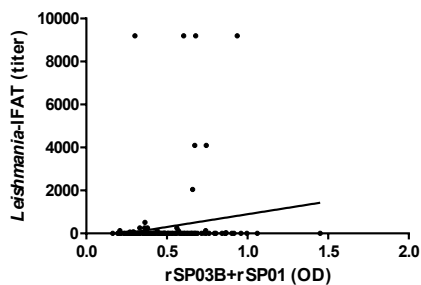

**H**  $r_s = 0.232 (0.095-0.361), p < 0.001$

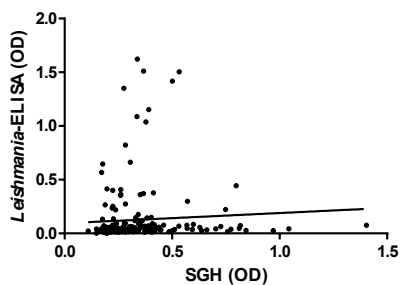

**I**  $r_s = 0.134 (-0.00-0.269), p = 0.053$

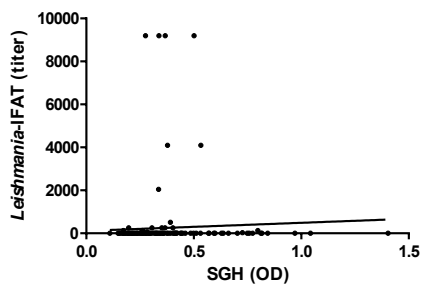

**J**  $r_s = 0.584 (0.483-0.669), p < 0.001$

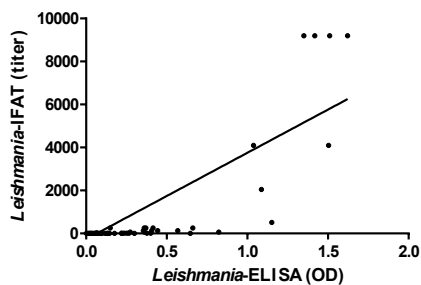

Supplement: Supplementary file 2 — Additional file 2: Figure S2. Correlations between serum antibodies levels at the end of sand fly season. a Between 43 KDa yellow-related protein (rSP03B) and rSP03B + 35.5 kDa apyrase (rSP03B + rSP01). b Between rSP03B and salivary gland homogenate (SGH). c Between rSP03B and Leishmania [enzyme-linked immunosorbent assay (ELISA)]. d Between rSP03B and Leishmania immunofluorescence antibody test (IFAT). e Between rSP03B + rSP01and SGH. f between rSP03B + rSP01 and Leishmania-ELISA. g Between rSP03B + rSP01 and Leishmania-IFAT. h Between SGH and Leishmania-ELISA. i Between SGH and Leishmania-IFAT. j Between Leishmania-ELISA and Leishmania-IFAT. Abbreviation: OD, optical density. [file 13071_2020_3993_MOESM2_ESM.pdf]
